# Annotation depth confounds direct comparison of gene expression across species

**DOI:** 10.1186/s12859-021-04414-y

**Published:** 2021-10-15

**Authors:** Elias Oziolor, Seda Arat, Matthew Martin

**Affiliations:** Computational Toxicology, Global Pathology and Investigative Toxicology, Worldwide Research and Development, Pfizer Inc, New York, USA

**Keywords:** RNAseq, TPM, Annotation, Cross-species comparisons, Pre-clinical species

## Abstract

**Background:**

Comparisons of the molecular framework among organisms can be done on both structural and functional levels. One of the most common top-down approaches for functional comparisons is RNA sequencing. This estimation of organismal transcriptional responses is of interest for understanding evolution of molecular activity, which is used for answering a diversity of questions ranging from basic biology to pre-clinical species selection and translation. However, direct comparison between species is often hindered by evolutionary divergence in structure of molecular framework, as well as large difference in the depth of our understanding of the genetic background between humans and other species. Here, we focus on the latter. We attempt to understand how differences in transcriptome annotation affect direct gene abundance comparisons between species.

**Results:**

We examine and suggest some straightforward approaches for direct comparison given the current available tools and using a sample dataset from human, cynomolgus monkey, dog, rat and mouse with a common quantitation and normalization approach. In addition, we examine how variation in genome annotation depth and quality across species may affect these direct comparisons.

**Conclusions:**

Our findings suggest that further efforts for better genome annotation or computational normalization tools may be of strong interest.

**Supplementary Information:**

The online version contains supplementary material available at 10.1186/s12859-021-04414-y.

## Background

Large part of the genetic code may be functional [1]. Exploring comparative genetic variability across species can generate hypotheses about the functional diversification across taxa [2], while transcriptomic profiles may provide a high-level view of the down-stream outcome of this diversity [3]. Whole transcriptome analysis has been used to understand evolutionary relationships and conservation of gene networks [4], as well as for comparison of the modularity of gene interactions across species [5]. On the other hand, functional cross-species comparisons can also be used to evaluate the predictive potential of pre-clinical species to model human molecular frameworks [6].

Comparisons of pre-clinical species as models of human functional genomics have been made, but rarely controlled on a large scale. A study by Lin et al. suggested that within species, tissues appear to have a more similar transcriptomic profile, compared to the same tissue across species [7]. However, Sudman et al. compared several other cross-tissue and cross-species datasets [3, 8], as well as a replicated sequencing from Lin et al., and suggested the opposite result [9]. The latter study, which features large-scale experiment batch control, reveals a potential for cross-species, cross-study comparability of functional genomic datasets for profiling tissue specific, evolutionarily conserved expression patterns. Later studies have confirmed that tissue specific gene expression is much more explanatory of the variation in expression than species wide differences [10].

Due to the unique variation in functional sequencing data [11], as well as the many sources of variation in quantification output, multiple methods have been developed to normalize these data for cross sample and study [12, 13]. Some methods have been found unreliable across experimental setup and developing technology [14, 15], while a few, such as transcript per million (*tpm*) and trimmed mean of M values (TMM), have been shown as more consistent between samples and possibly batches [14, 15]. On the other hand, direct cross-species comparisons of expression levels are rare, and researchers advise alignment to an orthologous chimeric transcriptome [16–18].

Direct comparisons of RNA expression across species can aim to inform appropriate pre-clinical model selection and ascertain human relevance of pre-clinical efficacy and safety signals. While transcriptional profile comparisons may elucidate tissue similarity in basal or active state, enabling direct comparison of RNA expression in an omic study may enable further resolution in identifying similarity in a specific set of potential targets. While creating a chimeric transcriptome may function sufficiently for pairwise comparisons [16, 17], it becomes computationally cumbersome and may reduce comparable gene sets substantially when more than two species are considered. For these reasons, we explored the possibility of mathematically normalizing, which may allow cross-species absolute RNA comparison of data derived from next-generation sequencing.

Cross-species comparison of RNA expression is complex due to a variety of confounding factors. Recently Zhou et al. [19] proposed a statistical approach for cross-species comparison of RNAseq data, called scale based normalization (SCBN), which built on hypothesis testing based normalization (HTN) [20]. This method identifies issues like the differences in gene lengths for orthologous genes between species, the difference in number of genes being mapped in orthologous gene sets. There Zhou et al., use a small set of orthologous genes to identify differential expression between species. While this approach introduces a sound correction for some of the differences between mouse and human biology, it doesn’t focus on the much wider annotation gaps exist in other pre-clinical species. Here we show that those annotation gaps, as expected, introduce a bigger set of biases in RNAseq comparisons.

In this study, we aim to evaluate current normalization techniques in providing robust comparisons of expression levels. We identify issues with *tpm* value comparison across species that appear to be related to annotation depth disparity and may stem from read mapping related to annotation differences. The disparity between human and pre-clinical transcriptome annotation includes but is not limited to (1) higher number of total transcripts found in human compared to other species; (2) higher transcript per gene ratio for each gene in humans; (3) larger identified paralog set in humans. Here we propose a simple workflow for normalization of *tpm* values that may enable direct abundance comparisons among human and other pre-clinical species, as well as provide a framework to identify potential pitfalls in direct human to pre-clinical species functional genomic comparison.

## Methods and results

We downloaded the latest annotations for human (*Homo sapiens*), cynomolgus monkey (*Macaca fascicularis*) (cyno), dog (*Canis familiaris*), rat (*Rattus norvegicus*), and mouse (*Mus musculus*) from Ensembl on May 9, 2019 (v.96). For each transcriptome, we combined coding and non-coding transcripts. We define depth of annotation as the number of unique transcripts present in the final dataset for each species.

### Overall depth of annotation evaluation

To test the effects of annotation depth disparity, we subdivided the human transcriptome at random into variable sizes (from 1 to 99% of the original transcriptome size in 1% increments). This approach allowed us to test, with high resolution, the effect for a variety of potential genome annotation depths and explore the magnitude and significance of impact for the preclinical species of interest. Before subdivision, we removed a set of 154 transcripts (from 10 genes that have variable *tpm* magnitude, in a stratified random sampling of bins between log values of 0.5 and 4.5, in the full transcriptome in the sample we selected described below). For each transcriptome size, we created 100 transcriptomes with random transcript composition. In each of the resulting 9,900 iterations we re-inserted the control 154 selected transcripts. We indexed the resulting transcriptomes using salmon quasi index with kmer size of 31 [21]. We selected a pancreatic sample (ERR315479) from the human protein atlas (HPA) [22] to align to all simulated genomes, and compared within and across tissue for depth and conforming transcriptional profile through dimensionality reduction. We quantified expression of that sample in each transcriptome using salmon [21] with default paired-end parameters with *validateMappings* selected. *Tpm* values were calculated on a transcript level using the default salmon quant methodology and introns were not considered for mappings.

**Fig. 1:**
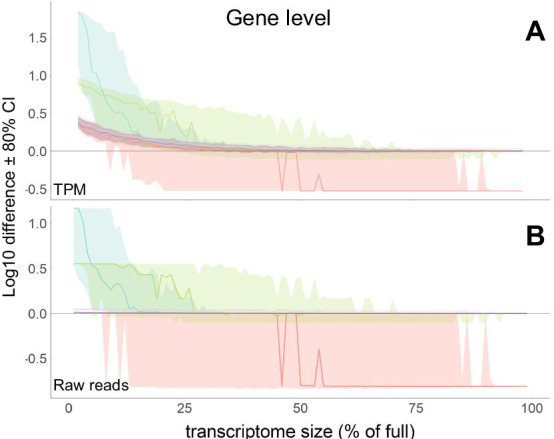
Transcripts per million (*tpm*) values for 10 sample genes appear inflated in sub-sampled transcriptomes, compared to their estimate in full length human transcriptome. **A** Gene level estimates of log10(tpm) difference for the 10 selected genes (individual lines and colors) from their estimate in the fully described human transcriptome. We selected genes through stratified random sampling from 10 bins of final abundance estimate across 9900 iterations of transcriptome with sizes from 1–99% (number of transcripts) of original transcriptome size. **B** Log10 difference in number of reads mapped to those genes in the same sampling scheme reveals that the large shifts in *tpm* estimates are driven by shifts in number of reads mapped. However, subtle and consistent shifts are independent of number of reads mapping to those genes

We imported transcript counts into R using tximport [23] package, summed the values to the gene level (using default tximport parameters) and plotted log differences between value in each sub-division and the full transcriptome in log10 of *tpm* values for each selected genes (Fig. 1A). When transcriptome sizes were subsampled, the *tpm* value of all test genes exceeded their calculated *tpm* value at the final transcriptome size (Fig. 1A). This relationship tended to be more pronounced when transcriptomes were less than 30% of original transcriptome size, and became less pronounced and became non-significant when transcriptomes were greater than 65% of original transcriptome size (Kruskal–Wallis test, Dunn’s *post-hoc*, Additional file 1: Fig. S1). The difference in *tpm* values can be explained by variable number of reads mapped for some of the transcripts (Fig. 1B), while other transcripts had no difference in number of mapped reads, suggesting that the difference in their *tpm* values is primarily based on a difference in the size of the transcriptome, rather than the success of the mapping.

### Drivers of *tpm* inflation

We calculated summed transcript per million values (denominator in Eq. 1 below) across the transcriptome for each quantification and plotted transcript *tpm* in relation to that value to show the relationship in the following *tpm* equation derivation:

1 $${tpm}_{i}=10^{6}\frac{{x}_{i}/{y}_{i}}{\sum_{j=1}^{n}{x}_{j}/{y}_{j}}$$

where x_i_ is the raw reads mapped to each transcript, y_i_ is the length of that transcript, and the denominator is the summation of that ratio across the all transcripts in the sample.

**Fig. 2:**
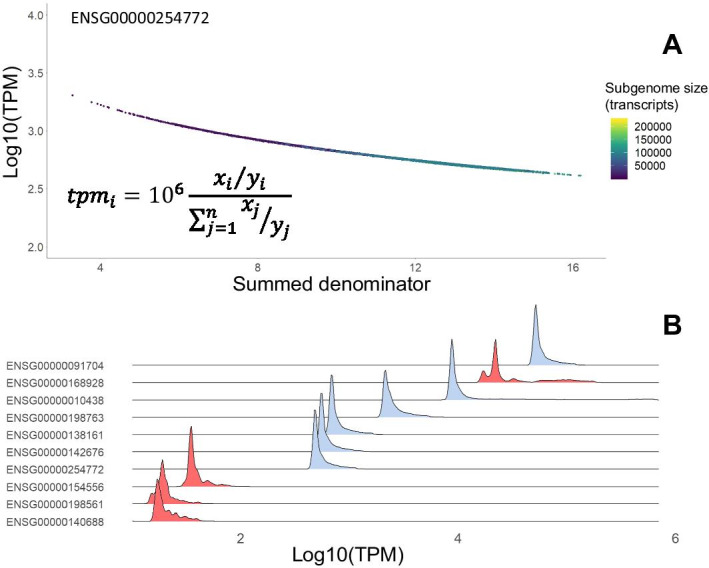
Inflation of *tpm* estimate in sub-sampled transcriptomes is consistently driven by variance in total number of reads mapped. **A** Consistent relationship between *tpm* estimate and total number of normalized reads is consistent with denominator decrease in shown equation for sub-sampled transcriptomes. This suggests that sub-annotated transcriptomes may suffer from similar inflation if samples are not corrected for total number of reads mapped. **B** Right skew *tpm* estimates of all examined selected genes reveals consistent inflation of the subsampled transcriptomes. Multi-modality of *tpm* values may be arising from competition for reads from paralogous targets only present in certain permutations of the sub-sampled transcriptomes (genes with distributions in red)

There is an inverse relationship between the size of the transcriptome and the calculated *tpm* value (Fig. 2), evident when the summed total per million values are plotted against the calculated *tpm* value for each of our test transcripts. The summed value represents the denominator of Eq. 1 and reveals a quantifiable and consistent relationship that is dependent on both the number of mapped reads, and may be related to the number of genes used in the summation, as a factor that affects the size of the denominator. These findings suggest that the inflation observed, and initially ascribed to variation in annotation depth, is simply a mathematical artifact of the total number of reads mapped in a sub-sampled genome.

### Effects of paralogs on *tpm* calculations

The set of 10 genes we chose to estimate across genome sizes contain a total of 421 paralogous transcripts in the human genome, defined by Ensembl. We removed those transcripts from the transcript set and re-built the set of subsampled transcriptomes as above.

We compared the *tpm* values for our selected genes on a gene level and show that excluding paralogs has a small, but noticeable impact on reducing competition for *tpm* value estimated for a particular gene (Additional file 4: Fig. S4A) or reads mapped (Additional file 4: Fig. S4B), and while it aids in reducing the multi-modality of *tpm* estimates for a few of the genes examined (Fig. 2B; Additional file 5: Fig. S5), it does not eliminate it. Overall, removing paralogs had little effect on the stratification of *tpm* values within similar size iterations. Thus, we suggest that the larger proportion of competition between read mappings occur on a transcript level, rather than gene level. This is consistent with the evolutionary distance between paralogs introducing enough variation in sequence that multi-mapping is minimal. This lack of competition lessens the concern that the higher number of paralogs in the human genome may serve to affect abundance estimates due to read map competition.

### Transcript to gene ratio influence

**Fig. 3:**
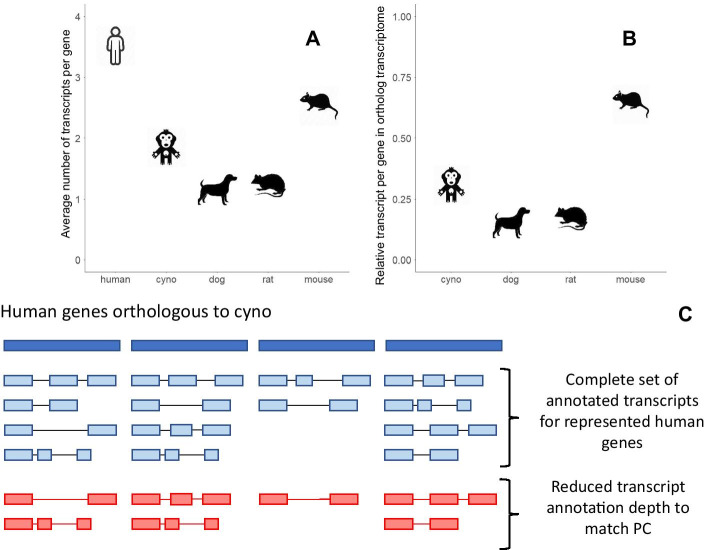
Disparity in transcriptome annotation between human and pre-clinical species. **A** Average amount of transcripts per gene is much higher in human than other pre-clinical species. **B** Transcripts per gene are even fewer when focusing on the genes orthologous among all species. **C** Testing set up, in which the human transcriptome was pre-clinicalized by subsetting the human transcriptome to the same number of transcripts per gene as found pairwise in pre-clinical species, iterating this process 100 times for each species and mapping the same human sample to all transcriptomes

Using the pancreatic sample from HPA mentioned above, we established the distribution of transcripts per gene for human, cyno, dog, rat and mouse from the existing coding and non-coding RNA sequences in Ensembl v.96 (Fig. 3A). We restricted the regions we compared to only gene identified as one to one orthologs across all species in the Ensembl database. When examining orthologous genes, the disparity in depth of annotation of transcripts is exacerbated (Fig. 3B).

As pre-clinical species contain from 20 to 75% the number of transcripts per gene for each orthologous gene with human, we wanted to understand the influence of the differential transcript annotation on final transcript and gene level estimated reads mapped and abundance. We generated a human transcriptome with one-to-one gene orthologs for each pairwise comparison with cyno, dog, rat and mouse. Thus, for each pairwise comparison, the human transcriptomes represent the same number of genes, but different number of transcripts from what a pre-clinical species would contain. For each human gene, we additionally reduced the number of annotated transcripts in the human transcriptomes, to the level found in the pairwise pre-clinical species. We iterated which transcripts we included 100 times at random with replacement. The result was 4 human transcriptomes with pairwise orthologous genes represented to each pre-clinical species and full annotation depth for human transcripts. In addition, 400 transcriptomes in which the transcript annotation depth was reduced in human, to the match number of transcripts found in each pairwise pre-clinical species (Fig. 3C), which we will refer to as pre-clinicalized human transcriptomes.

**Fig. 4:**
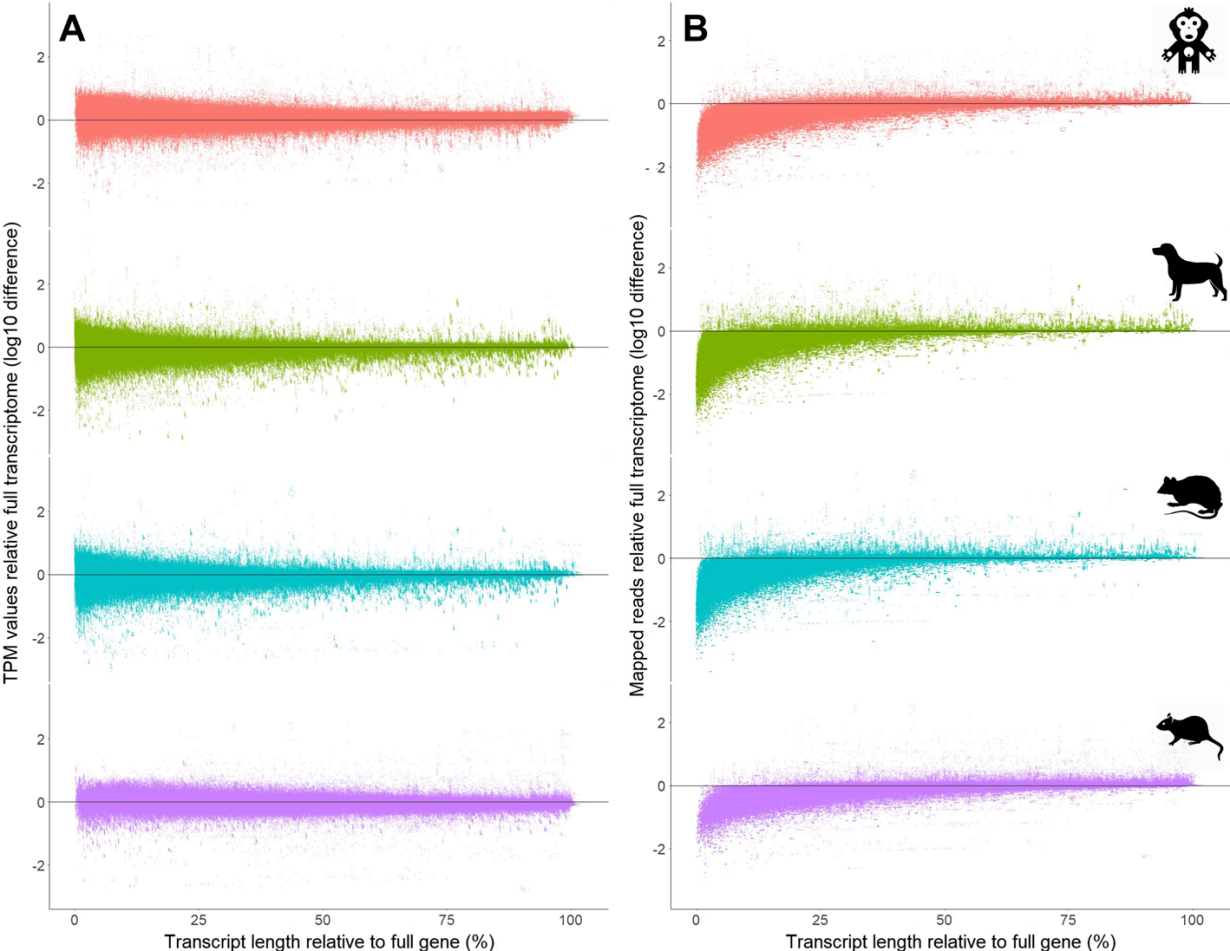
Pre-clinicalized transcriptomes reveal pattern of read mapping reduction and mean *tpm* stability. **A** *tpm* estimates remain stable for genes, even when small percentage of total transcript length is included in pre-clinicalized transcriptomes. **B** This is true even on the background of a reduced number of reads mapped genes with very little transcript length represented

We explored the differences in gene-level estimates of raw mapped reads and abundance between full representation transcriptomes versus reduced transcript annotation representation and show reduction on final gene *tpm* values in most species (Additional file 11: Fig. S11A). This is paired with a clear reduction of total mapped reads per gene in pre-clinicalized transcriptomes (Additional file 11: Fig. S11B). The reduction of reads mapped tends to occur in genes, which have less transcript length represented in the pre-clinicalized transcriptomes (Fig. 4). Due to the control for reads mapped per unit length in the *tpm* calculation, the reduction in reads mapped in underrepresented genes does not lead to a systematic alteration in *tpm* estimates for those genes, while it does show increased variability in *tpm* values at lower lengths.

**Fig. 5:**
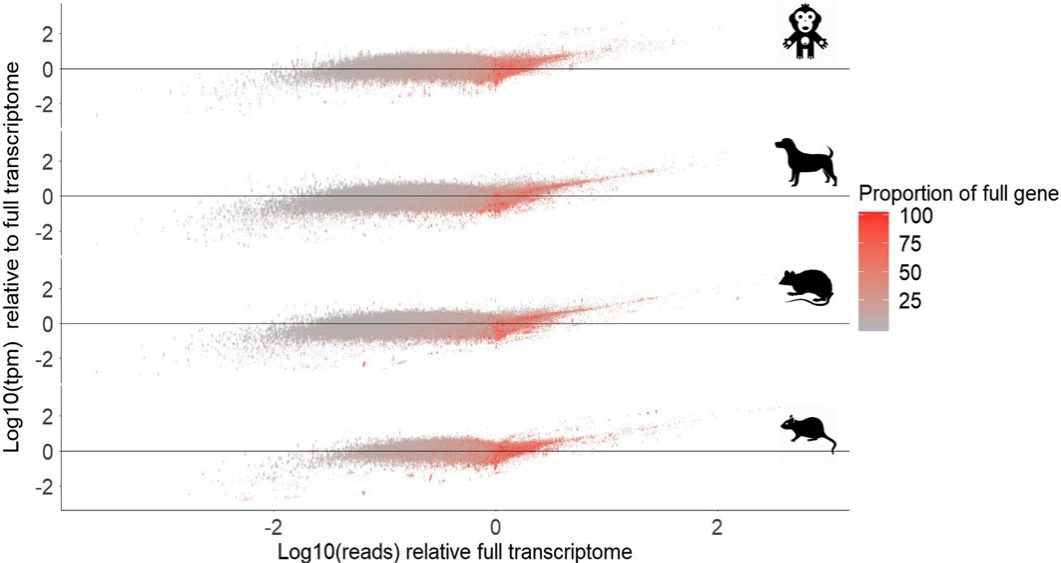
Estimates of *tpm* are relatively robust to reduction in reads mapped per gene, while read mapping increases lead to proportional *tpm* inflation. Few genes with high proportion of their length of transcripts represented present with higher reads mapped compared to full representation transcriptome. This could be due to lower competition for reads from similar transcripts in paralogous genes that are poorly annotated in pre-clinical species. This inflation of mapped reads leads to inflation of *tpm* estimates, suggesting a potential issue in direct abundance comparisons between species with vastly divergent genome annotations

On the other hand, there is a clear over-estimation of *tpm* values for a specific set of genes in pre-clinicalized human transcriptomes (Fig. 5). A set of genes, well represented by transcript length, tend to have more reads mapped to them in pre-clinicalized transcriptomes compared to full size transcriptome. Those genes also tend to have an inflated *tpm* estimate, tending to correlate with reads mapped (Fig. 4). We explored the expression of those genes in the full transcriptome compared to 1000 random samples of the same size and show that the genes that are over-represented in pre-clinicalized transcriptomes tend to be ones of lower expression (Additional file 12: Fig. S12A). Further, size of these over-represented genes, does not seem to be consistently lower or higher than a random sample among species (Additional file 12: Fig. S12B). These findings suggest that when transcriptomes are reduced to pre-clinical species annotation sizes, a set of competitor transcripts are removed, allowing for higher mapping of reads to low expression genes and leading to inflated *tpm* estimates.

To further characterize the genes that tend to have over-estimated expression, we extracted those genes among species and plotted all their occurrences (Additional file 13: Fig. S13A). The instances, in which those genes become over-represented compared to final sample are only when a large proportion of the gene’s length is represented (i.e. more of the longer transcripts). In such instances, more reads tend to map to these genes, compared to full human transcriptome, likely due to the lack of competition from transcripts of other similar genes (Additional file 13: Fig. S13A). This manifests as a positive relationship between difference in *tpm* value, due to higher read count for those genes. However, this relationship is not present in the other set of genes (Additional file 13: Fig. S13B), which do not tend to be over-estimated at higher gene representation. Those also tend to be genes with higher gene expression (Additional file 12: Fig. S12).

### Ensembl historical annotation of human

To test if the pre-clinicalization of the human transcriptome is reflective of the historical progression of human transcript annotation, we downloaded historical annotation of the human transcriptome throughout versions 43 to 97 of Ensembl (July 8th, 2019). We mapped our human sample and examined the difference in *tpm* estimates to the genes in the first human transcriptome annotation throughout the progression of adding more transcripts in later versions of the transcriptome.

**Fig. 6:**
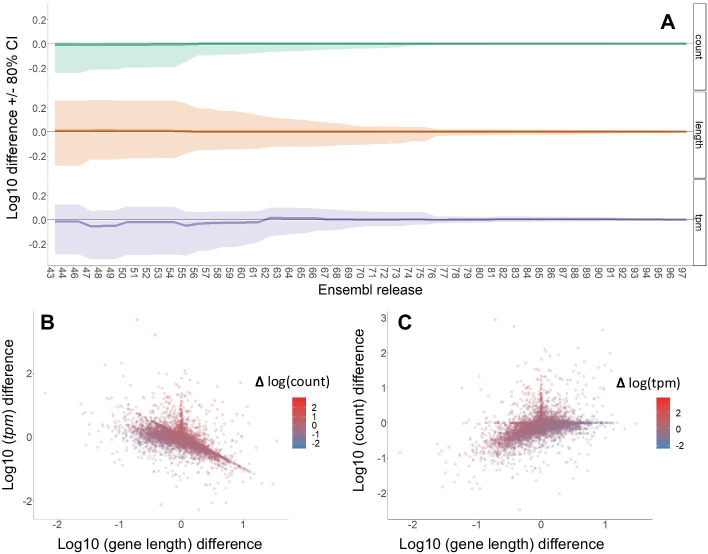
Historical variation in human transcriptome annotation leads to variable abundance estimates for the same genes in the same sample. **A** Abundance and gene length variation in human transcriptome annotation are accompanied with better read mapping over time, consistent with the improvement of the representation of the human transcriptome. **B** Higher gene length in earliest examined version (v43) is consistent with lower *tpm* estimates, while this relationship is less consistent for genes with shorter length. **C** This reduced *tpm* is driven by the lack of increase in reads mapping to longer versions of current genes, which suggests that those lengths were artificially adding improper sequence, which was not part of expressed portion of the gene

When examining genes conserved among all transcriptome annotations, we observed a trend of varying *tpm* estimates for a large set of genes that were annotated in the original transcriptome (Fig. 6A). Estimates of *tpm* values in historic versions of the genomes were distributed around the mean estimates for the same genes in the current latest genome (v.97). However, mapped read counts were consistently lower in previous versions of the Ensembl human annotation, suggesting that the current assembly is better representative of the true genetic code in the expressed portion of the human genome.

Reduced *tpm* estimates likely stem from inflated gene length, which does not result in higher read content (Fig. 6B). We examined that in release 43 of the human genome, while mapped reads for each gene tended to be lower, gene length varied, with some genes having estimated lengths of twice or more of their size in the most current assembly (Fig. 6A). We quantified our sample as indicated in previous sections. We observe that *tpm* decreases in strong relationship with gene length (Fig. 6B), while it does not necessarily hold that relationship for genes with shorter than current length. We removed genes with higher gene length in earlier assemblies, we reconstitute the inflated *tpm* values that we observe in reduced size transcriptome mapping (Additional file 14: Fig. S14A). This may be due to either lower number of reads mapped and reduced gene length. When observed over time, the genes with higher gene length in earlier assemblies vary strongly even until most recent transcriptomes (Additional file 14: Fig. S14B). One potential explanation could be that genes with longer length in older assemblies, have formerly received lower number of reads mapped in older annotations or assemblies (Fig. 6C). The wide variation in gene length across time assemblies and its profound impact in *tpm* values within the same reference genome raise a caution in the comparison of *tpm* estimates across species.

### Correction of *tpm* values with experimental data across species

To verify that *tpm* is inflated due to sequencing depth and not annotation differences, we downloaded publicly available datasets across species. Between tissue differences in transcriptomic profile are smaller than those between species [9, 10]. We used human transcriptomic data from GTEx (v7), human protein atlas (HPA) and data for four pre-clinical species (cynomolgus monkey, dog, mouse and rat) from Naqvi et al. [10]. These data included 13 commonly sampled organs leveraging the range observed in Naqvi et al. [10] (lung, adrenal gland, brain, skin, liver, testis, colon, heart, pituitary gland, spleen, skeletal muscle, thyroid gland, adipose tissue). GTEx and pre-clinical species data was taken as *tpm* values from publications, while HPA data was run through the salmon pipeline (as described above).

**Fig. 7:**
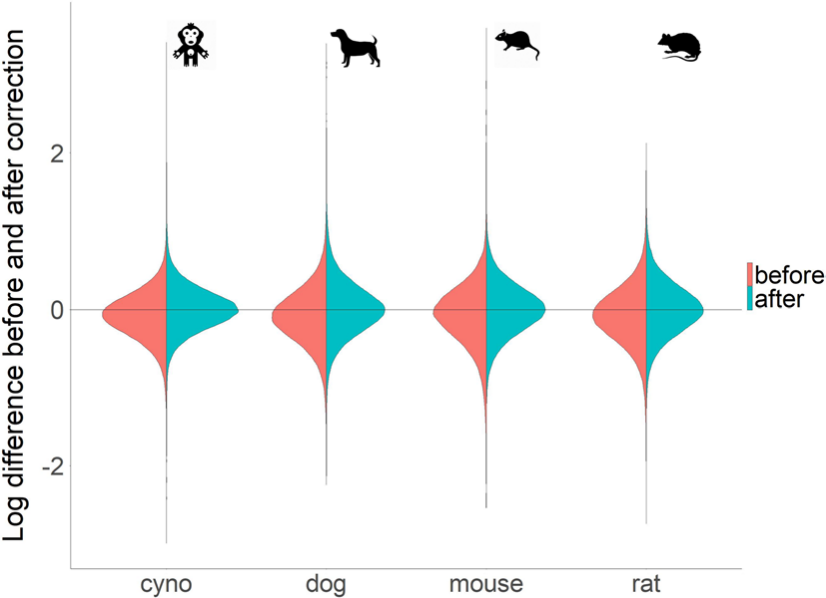
Trimmed Means of M values (TMM) correction of experimental data removes skew in pre-clinical species, stemming from variation in number of reads mapped. Within tissue comparisons of *tpm* values for 13,000 orthologous genes to their median tissue value in human (GTEx and HPA) reveal a consistently lower average *tpm* in pre-clinical species. This is likely driven by higher depth of sequencing in pre-clinical datasets. TMM accounts and corrects for differences in sequencing depth

Given the known similarity of expression profiles within organs between species, we aimed to observe gene level *tpm* disparity between human and pre-clinical species on a per tissue level. We calculated median *tpm* value for a set of orthologous genes across all species (~ 13,000). We further subtracted log10(*tpm*) values for each sample of pre-clinical species from the median human values within organ and plotted the differences (Fig. 7).

The mean and median *tpm* values in pre-clinical species were consistently lower than human. This is contrary to the expectation for the direction of difference, driven by genome annotation depth (as examined in Fig. 1). However, this is clearly in line with a relationship between *tpm* values, driven by depth of sequencing (Additional file 2: Fig. S2). To remedy this technical disparity, driven by total number of mapped reads, we applied a TMM normalization, using the NOISeq package [24], on orthologous set of genes, with expression values above 1 *tpm*. This normalization shifts distributions of differences from human to approximately 0 and enables direct comparison of abundance values on the orthologous gene set. The code/package information is available in Additional file 16.

We followed up these analyses with a targeted approach, using a previously proposed set of human housekeeping genes [25]. Of the 3804 proposed human housekeeping genes, 1904 had orthologs across all pre-clinical species. The TMM correction, while performed on all genes, successfully improved the comparability of absolute expression estimates of pre-clinical species to human as well (Additional file 6: Fig. S6). Exploring the variability in correction on an tissue level reveals that many organs receive minimal correction within a species, while others were barely corrected (Additional file 6–9: Figs. S6–9). The distributions of these housekeeping genes in most tissues look very similar to each other within the species, it is because of housekeeping genes expected to be expressed across all tissues to maintain basic cell functions. The distribution similarities become more obvious “after” TMM correction across all tissues/pre-clinical species, supporting the argument that TMM correction is needed for direct comparison between different species.

## Discussion

Analysis of transcriptional variation is crucial in understanding the molecular machinery of organisms [10]. Drawing inferences about functional contrasts between species is in the heart of understanding evolutionary processes, but also more applied tasks, like drug safety species selection and understanding human relevance of target-driven efficacy and safety signals. In this manuscript we identify potential issues with direct comparison of transcriptomic data across species with wide annotation gaps. We focus on the differences between four commonly used pre-clinical species and how those differences impact the *tpm* values, commonly used for direct expression comparison. Here we suggest a straightforward workflow of *tpm* estimation followed by TMM normalization to enable direct abundance comparisons among species. In addition, we iterate the magnitude of existing issues in paralogous transcript read map competition, annotation depth and gene length variation across species, which still remain issues in enabling direct comparisons. Here we focus on salmon as our mapping algorithm, and while some of these results may vary across others available in the space, the salmon is among the most preferred algorithms and it performs similarly to other popular mapping tools [26].

Regulatory bodies aim to evaluate the predictivity and fit of a pre-clinical species used for pharmaceutical safety or efficacy evaluation. The justification of species selection must be based on functional concordance with human molecular responsiveness. Often a one to one comparison is attempted in basic functional end-points, such as whole transcriptome sequencing derived gene expression. However, as we see in this study, abundance estimates on a per gene level may vary between experiments or species. We present two large potential issues for one to one functional comparisons of transcriptomic data between humans and pre-clinical species: (1) depth of genome annotation; (2) competition for read mapping in low expression and poorly annotated gene families. A large majority of the first issue appears to be resolved by correction for the total reads mapped per sample (TMM normalization of *tpm* values). We suggest that such workflow may bring abundance estimates to more comparable levels, by correcting for the variation in *tpm* due to variation in total reads mapped.

The effort for more directly comparable gene expression values between species must revolve around equalizing conditions in the computational process. As an example, our simulations suggest that focusing on a set of genes homologous across all species (Fig. 4–7), may yield rather robust *tpm* estimates, with a few exceptions. The use of TMM correction on *tpm* values [27], assumes that majority of genes are not differentially expressed, which is consistent with the idea that tissue specific expression is more similar between species than between tissues [9, 10]. While differential expression between species has been shown to be of lower magnitude than between tissues [10], evolutionary divergence still is identifiable through level of difference in expression across further evolutionarily distant species. We also see that even within the same families, there can be huge variation in genome sizes, which could further contribute to a variation in genome differentiation [28]. Evolutionary pressures have also imposed selective forces on expression pathways across mammals, which can be considered in direct comparisons [27]. Thus, the usage of TMM values should be used with caution in species with long evolutionary separation. Others use PCA value normalization [10], which also may allow for a level of comparability, in which there is control for transcriptome size, mapped reads and other factors which may skew expression in pre-clinical species compared to human. Some have proposed a normalization based on gene length corrected trimmed mean of M-values (GeTMM) for inter-sample and intrasample comparisons, that is also based on *tpm* and TMM [29]. GeTMM was shown to work better in human cancer samples over other normalization techniques, yet it has not been tested its capability for intraspecies comparisons. Thus, we recommend such attempts to normalize datasets with caution and robust documentation of the normalization and comparison processes.

The second issue remains: there are still a set of genes, which have normally low expression in full human transcriptome, but when reduced to their diversity in pre-clinical species, exhibit an artificially inflated abundance estimate. We suggest that this disparity is due to a removal of set of genes, annotated in human, but not yet discovered in pre-clinical species. This removal reduces the competition for read mapping in poorly annotated gene families in pre-clinical species and artificially inflates the abundance estimates for otherwise low expression genes. Using an orthologous subset genome is likely not the preferrable option biologically. Given that only ~ 13,000 genes have proper orthologs when evolutionarily distant species are considered (dog, rodents), this heavily limits the biological interpretation of results of differential expression with a orthologous genome used for quantitation. We argue that using such a work-around is not fixing the problem, but rather avoiding it.

Paired with the problem of ortholog abundance is the divergence of gene length among orthologous genes between species (Additional file 12: Fig. S12). Even within the human genome, lengths of genes vary widely among releases (Additional file 13: Fig. S13B). The human gene lengths varied more in the earlier versions as annotations were improving drastically (Additional file 14: Fig. S14B). While they still vary between current versions, that absolute variability is lower. A trend alike early human annotations can be seen in some preclinical species. An example of that is the doubling of transcripts available for the dog transcriptome between Ensembl versions 102 and 104. Additionally, efforts like the Earth BioGenome [30] project and the Vertebrate Genome Project [31] are making incredible strides in resolving issues around reference genome annotation and assembly. The *tpm* correction factors in gene length, which makes expression values among genes comparable, but if the annotation of the matching ortholog between species has divergent gene lengths due to technical and not biological reasons, this will pose a confounding factor for direct abundance comparisons.

## Conclusions

There are many issues, both biological and technical, in the direct comparison of whole transcriptome RNA sequencing data directly between species. With this manuscript we hope to introduce some of the questions we have found to influence this comparison and offer some corrective measures. Despite that, functional comparison with pre-clinical species is still an essential metric for species selection and translation, which necessitates a body of work that would permit better confidence for regulatory and scientific purposes. Our main suggestion is a heightened attention to fully annotating pre-clinical species. Such an effort would allow us to understand both evolutionary and technical constraints to direct transcriptomic comparisons, as well as aid in better safety assessment by using the most relevant species for each evaluated target.

## Supplementary Information

**Additional file 1: Figure S1** Inflation of *tpm* values for genes is significant up to 65% of original genome size. A Kruskal-Wallis test with Dunn’s post-hoc shows that p-values adjusted for multiple testing (Benjamini-Hochberg) are below 0.05 in genomes that are less than 65% of original genome size.

**Additional file 2: Figure S2** Increases in relative normalized read counts per sample are consistent with reduction of *tpm* value across all examined orthologous genes. We examined all samples from Naqvi et al. [10], the GTEx and HPA datasets and focused on orthologous genes. We calculated the sum of gene length normalized raw reads per sample and related them to the most highly sequenced sample. We show a negative relationship (linear model) between log10(*tpm*) per gene values and total reads mapped across the entire orthologous transcriptome.

**Additional file 3: Figure S3** Transcript competition for reads presents as multi-modality in *tpm* estimation across sub-sampled transcriptomes. A) Many of the examined test transcripts present with a stratification in their abundance estimate, even at the same total read count per sample. B) Some of this multi-modality is rescued when *tpm* are summed to the gene level. This suggests that the stratification is present due to a competitor transcript of the same gene acquiring reads in some cases and not in others. This leads to lower *tpm* estimate of test transcript when competitor is present, but no difference when transcripts are summed to gene level.

**Additional file 4: Figure S4** Removal of paralogous transcripts does not lead to an appreciable inflation in *tpm* in the same setup. Repetition of setup from Fig1 with the paralogous transcripts removed reveals the same relationship between gene-wise A) *tpm* levels and B) read counts across test transcripts in sub-sampled transcriptomes.

**Additional file 5: Figure S5** Multi-modality of gene-level estimates of *tpm* is lower, but still present when paralogous transcripts are removed.

**Additional file 6: Figure S6** Distribution of 1,904 housekeeping and orthologous genes’ expression log difference before Trimmed Means of M values (TMM) correction and after TMM correction in pre-clinical species relative to human. The labeled values are the median of the distributions before and after TMM correction. The median of TMM corrected distribution is closer to 0, meaning that TMM correction makes the pre-clinical species distributions more comparable to human.

**Additional file 7: Figure S7** Distribution of 1,904 housekeeping and orthologous genes’ expression log difference before Trimmed Means of M values (TMM) correction and after TMM correction across 13 tissues in cyno relative to human. The labeled values are the median of the distributions before and after TMM correction. The median of TMM corrected distribution is closer to 0, meaning that TMM correction makes the pre-clinical species distributions more comparable to human.

**Additional file 8: Figure S8** Distribution of 1,904 housekeeping and orthologous genes’ expression log difference before Trimmed Means of M values (TMM) correction and after TMM correction across 13 tissues in dog relative to human. The labeled values are the median of the distributions before and after TMM correction. The median of TMM corrected distribution is closer to 0, meaning that TMM correction makes the pre-clinical species distributions more comparable to human.

**Additional file 9: Figure S9** Distribution of 1,904 housekeeping and orthologous genes’ expression log difference before Trimmed Means of M values (TMM) correction and after TMM correction across 13 tissues in mouse relative to human. The labeled values are the median of the distributions before and after TMM correction. The median of TMM corrected distribution is closer to 0, meaning that TMM correction makes the pre-clinical species distributions more comparable to human.

**Additional file 10: Figure S10** Distribution of 1,904 housekeeping and orthologous genes’ expression log difference before Trimmed Means of M values (TMM) correction and after TMM correction across 13 tissues in rat relative to human. The labeled values are the median of the distributions before and after TMM correction. The median of TMM corrected distribution is closer to 0, meaning that TMM correction makes the pre-clinical species distributions more comparable to human.

**Additional file 11: Figure S11** Both abundance and read mapping are reduced in pre-clinicalized human transcriptomes. A) *tpm* estimates are on average lower in pre-clinicalized transcriptomes for species, other than cyno. B) This is congruent with left skew in read counts compared to fully represented human transcriptome.

**Additional file 12: Figure S12** Inflation of *tpm* values in pre-clinicalized transcriptomes happens in genes with lower than average expression, while it is not related to gene length. A) Random subsampling of gene expression values (1000 permutations) of the same size as number of genes as ones found to have inflated *tpm* values in pre-clinicalized transcriptomes. The average expression in sampled genes is markedly higher for all species, compared to the average expression in the genes found to have inflated *tpm* in pre-clinicalized transcriptomes (black vertical bars). B) Same sampling did not yield a consistent difference between gene length of inflated *tpm* genes and the rest of the transcriptome.

**Additional file 13: Figure S13** Genes with inflated *tpm* values in pre-clinicalized transcriptome only experience such inflation, when large proportion of the total transcript length is present. A) Reveals genes that were shown to experience inflation in *tpm* when examined in a pre-clinicalized transcriptome. B) Same relationship for all other genes shows that inflation does not occur even when at high proportions of transcript length present.

**Additional file 14: Figure S14** Historical variation in gene length plays a role in *tpm* estimates. A) In historical ensemble human transcriptome releases, genes that did not have inflated gene lengths have *tpm* estimates more consistent with ones from current day human transcriptome. B) Gene length variation in genes with historically inflated length estimates is highly variable even until very recent releases.

**Additional file 15** HPA pancreas quantitation.

**Additional file 16** Collated code.

## Data Availability

All data used for this manuscript are publicly available and their origin is noted in the code for the article. The code is also public and is hosted at a GitHub repository (https://github.com/eoziolor/annotation_tpm_comparison).

## Abbreviations

TPM: Transcript per million
TMM: Trimmed mean of M values
SCBN: Scale based normalization
GTEx: Genotype-tissue expression
HPA: Human protein atlas
GeTMM: Gene length corrected trimmed mean of M-values
