# Supplementary figures and images for: Annotation depth confounds direct comparison of gene expression across species
Source: BMC Bioinformatics. 2021 Oct 15;22:499. doi: 10.1186/s12859-021-04414-y (PMC8518172; doi:10.1186/s12859-021-04414-y)

## Slide 1
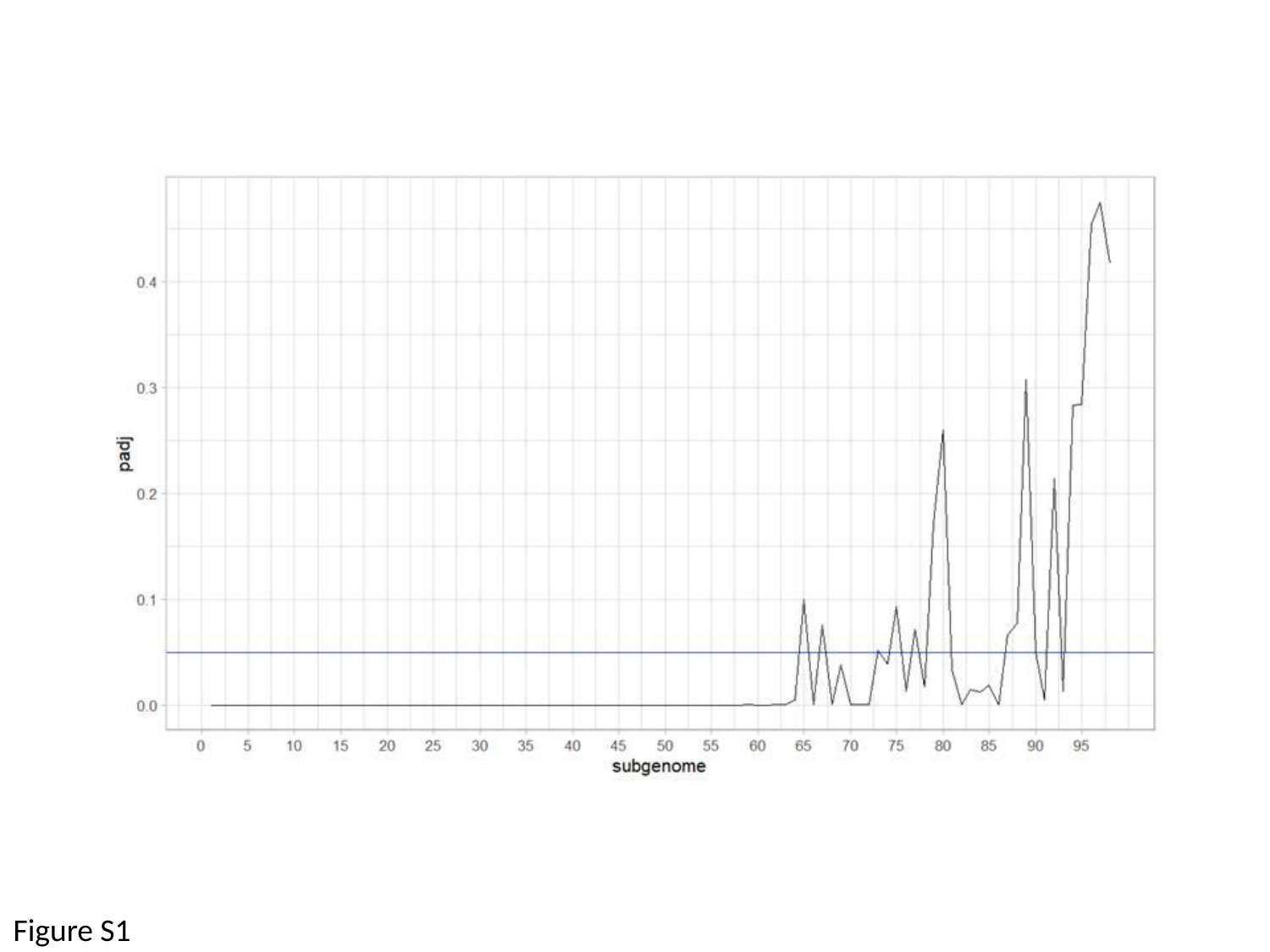

Figure S1

Supplement: Supplementary file 1 — Additional file 1: Figure S1 Inflation of tpm values for genes is significant up to 65% of original genome size. A Kruskal-Wallis test with Dunn’s post-hoc shows that p-values adjusted for multiple testing (Benjamini-Hochberg) are below 0.05 in genomes that are less than 65% of original genome size. [file 12859_2021_4414_MOESM1_ESM.pptx]

## Slide 1
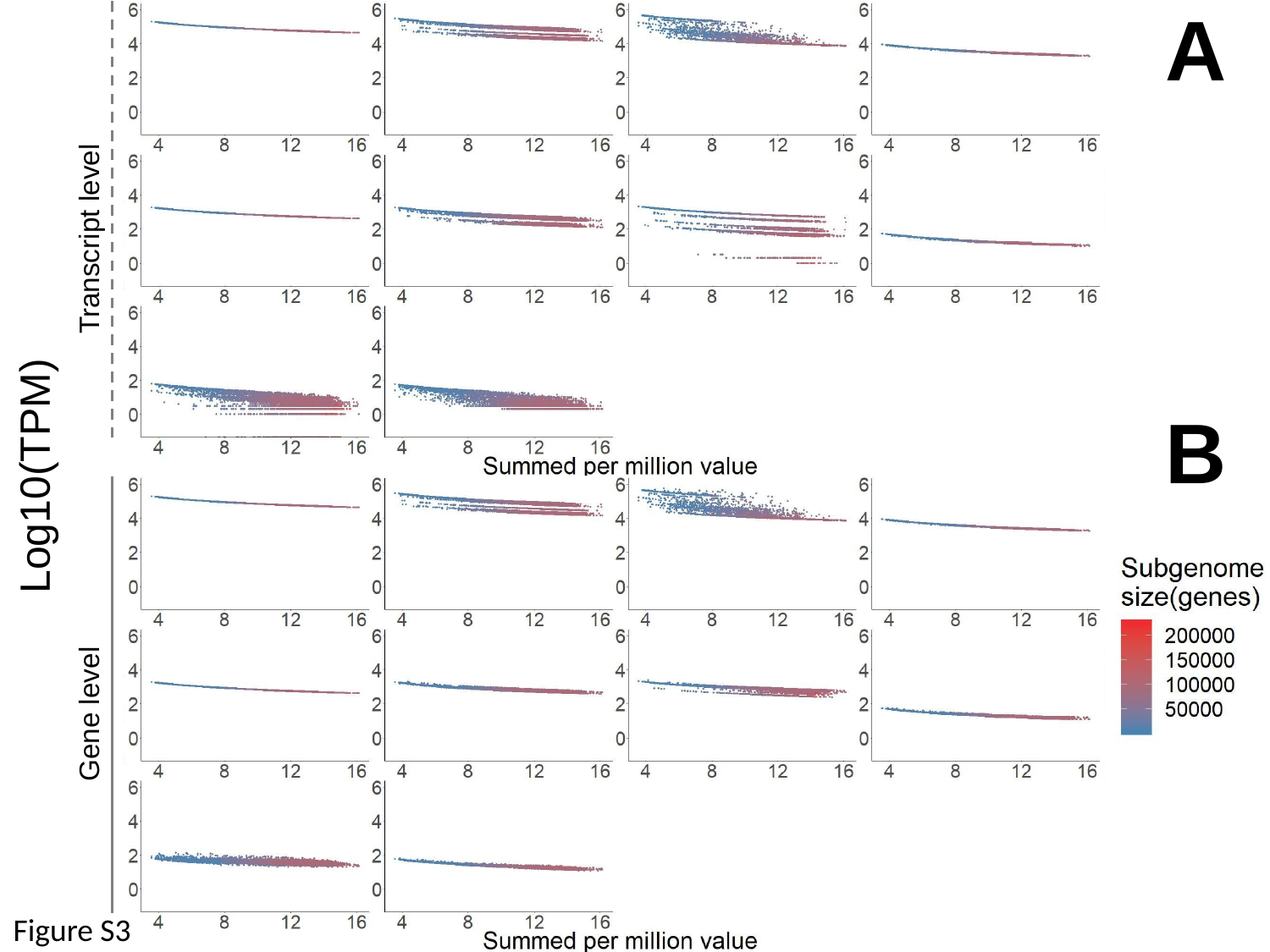

A
Transcript level
B
Log10(TPM)
Gene level
Figure S3

Supplement: Supplementary file 3 — Additional file 3: Figure S3 Transcript competition for reads presents as multi-modality in tpm estimation across sub-sampled transcriptomes. A) Many of the examined test transcripts present with a stratification in their abundance estimate, even at the same total read count per sample. B) Some of this multi-modality is rescued when tpm are summed to the gene level. This suggests that the stratification is present due to a competitor transcript of the same gene acquiring reads in some cases and not in others. This leads to lower tpm estimate of test transcript when competitor is present, but no difference when transcripts are summed to gene level. [file 12859_2021_4414_MOESM3_ESM.pptx]

## Slide 1
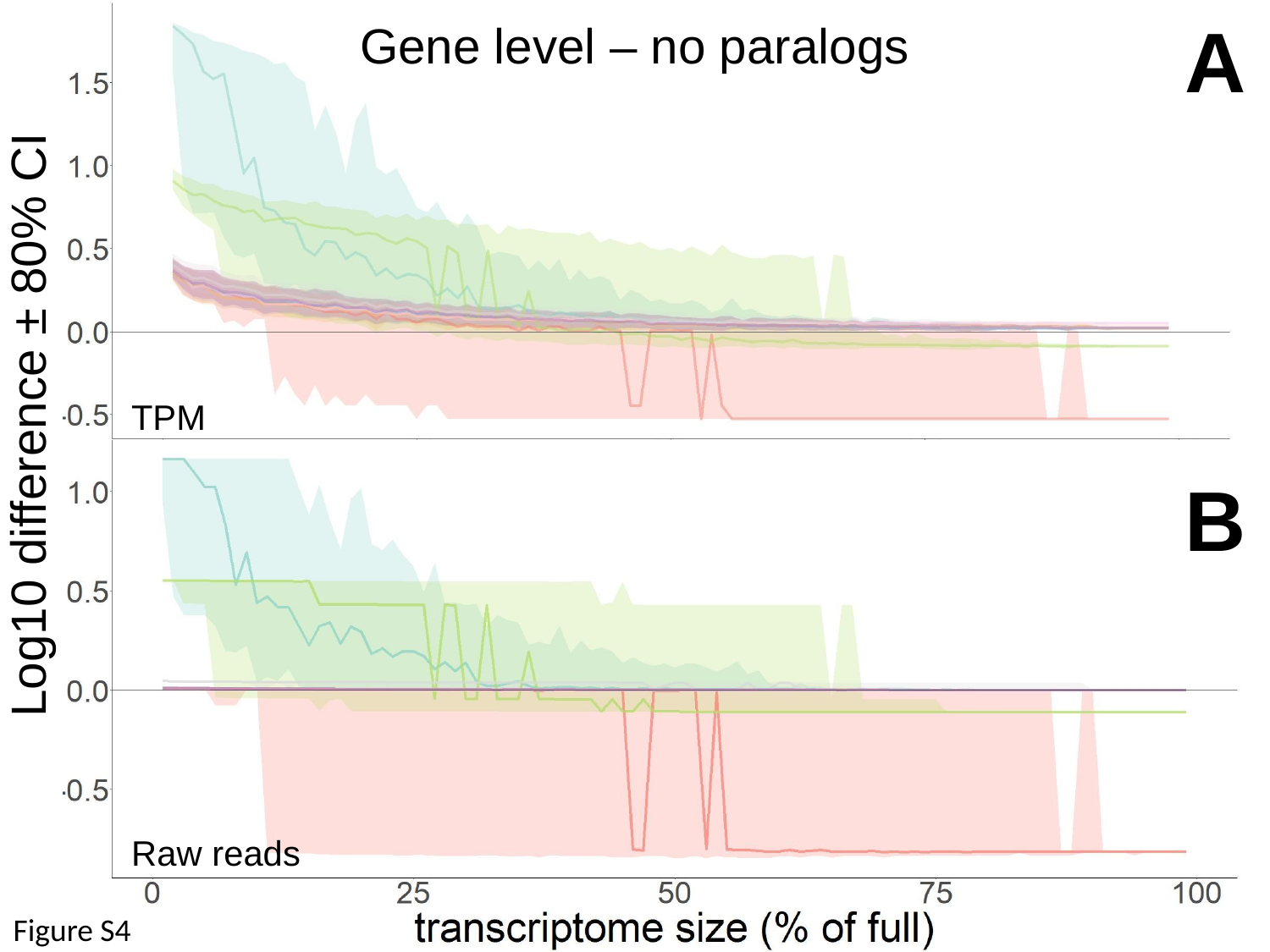

A
Gene level – no paralogs
TPM
Log10 difference ± 80% CI
B
Raw reads
Figure S4

Supplement: Supplementary file 4 — Additional file 4: Figure S4 Removal of paralogous transcripts does not lead to an appreciable inflation in tpm in the same setup. Repetition of setup from Fig1 with the paralogous transcripts removed reveals the same relationship between gene-wise A) tpm levels and B) read counts across test transcripts in sub-sampled transcriptomes. [file 12859_2021_4414_MOESM4_ESM.pptx]

## Slide 1
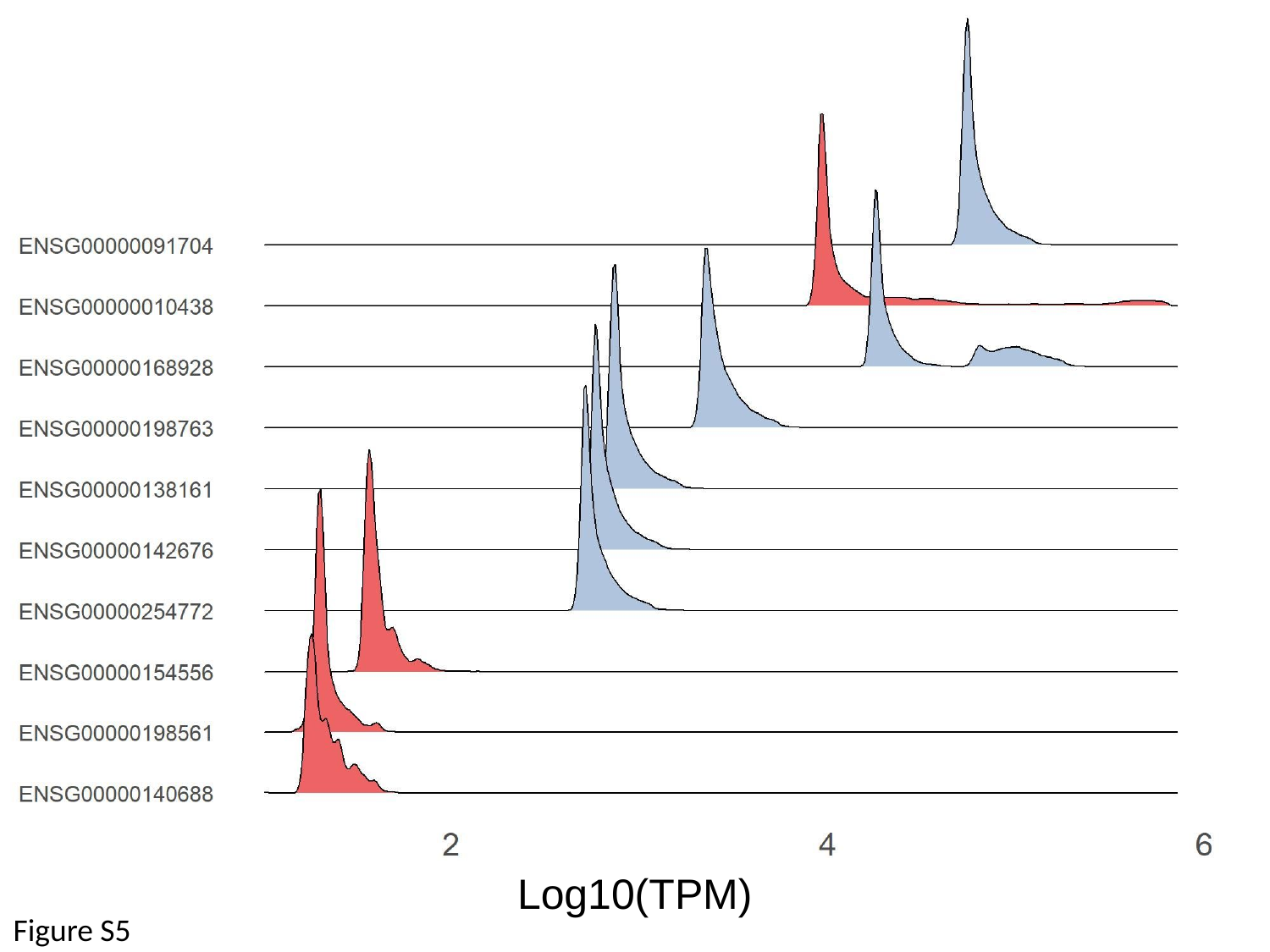

Log10(TPM)
Figure S5

Supplement: Supplementary file 5 — Additional file 5: Figure S5 Multi-modality of gene-level estimates of tpm is lower, but still present when paralogous transcripts are removed. [file 12859_2021_4414_MOESM5_ESM.pptx]

## Slide 1
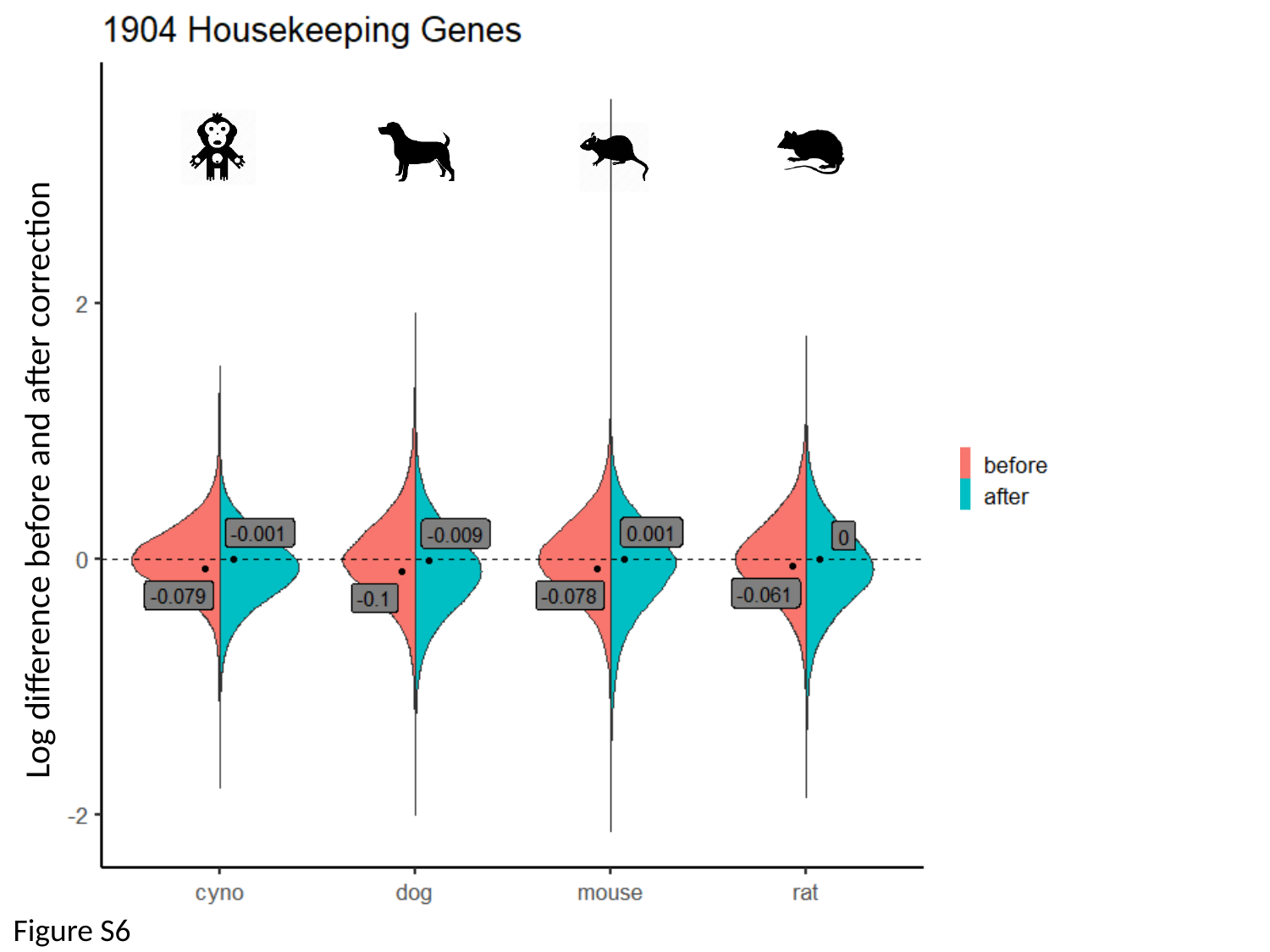

Log difference before and after correction
Figure S6

Supplement: Supplementary file 6 — Additional file 6: Figure S6 Distribution of 1,904 housekeeping and orthologous genes’ expression log difference before Trimmed Means of M values (TMM) correction and after TMM correction in pre-clinical species relative to human. The labeled values are the median of the distributions before and after TMM correction. The median of TMM corrected distribution is closer to 0, meaning that TMM correction makes the pre-clinical species distributions more comparable to human. [file 12859_2021_4414_MOESM6_ESM.pptx]

## Slide 1
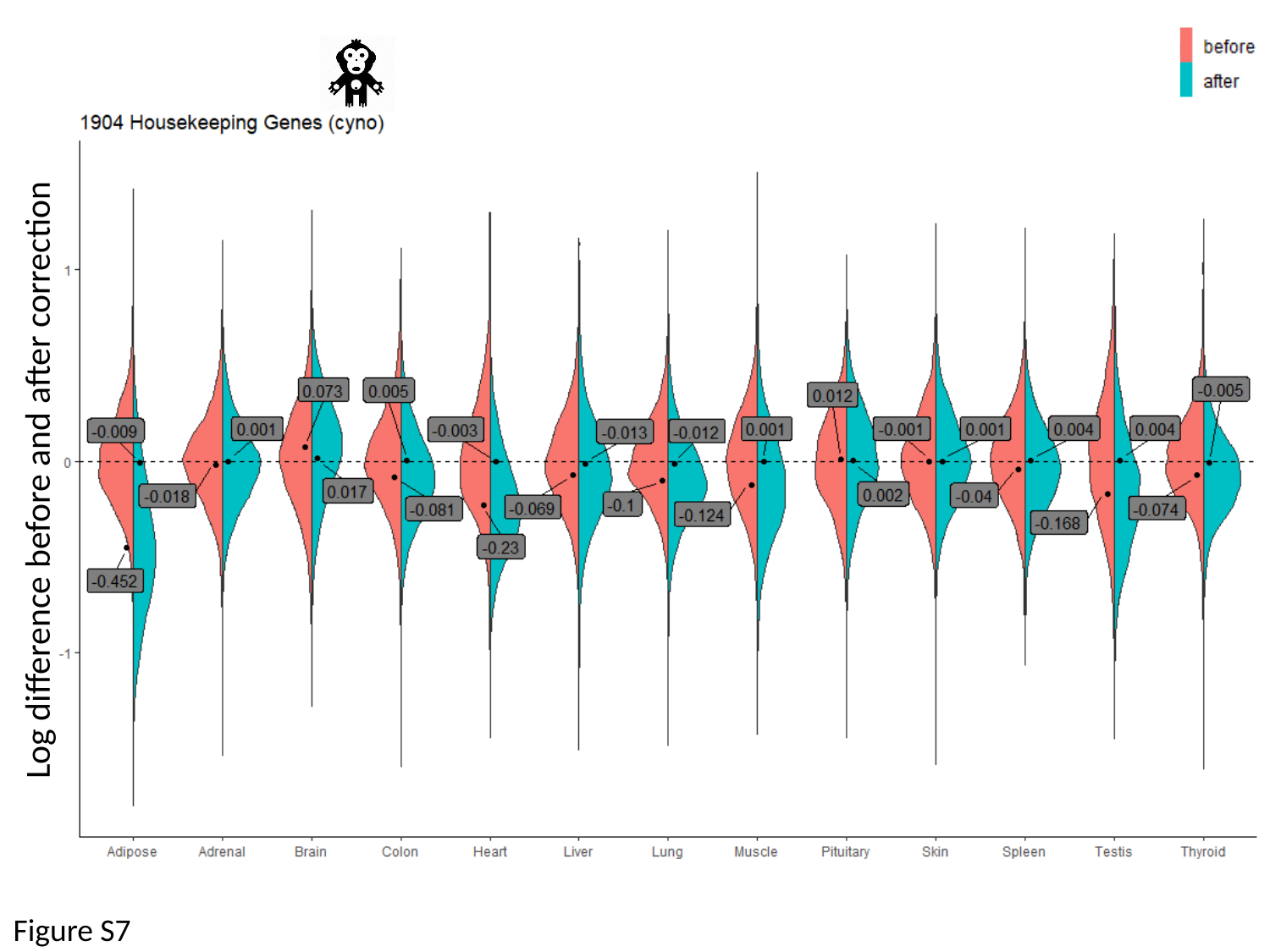

Log difference before and after correction
Figure S7

Supplement: Supplementary file 7 — Additional file 7: Figure S7 Distribution of 1,904 housekeeping and orthologous genes’ expression log difference before Trimmed Means of M values (TMM) correction and after TMM correction across 13 tissues in cyno relative to human. The labeled values are the median of the distributions before and after TMM correction. The median of TMM corrected distribution is closer to 0, meaning that TMM correction makes the pre-clinical species distributions more comparable to human. [file 12859_2021_4414_MOESM7_ESM.pptx]

## Slide 1
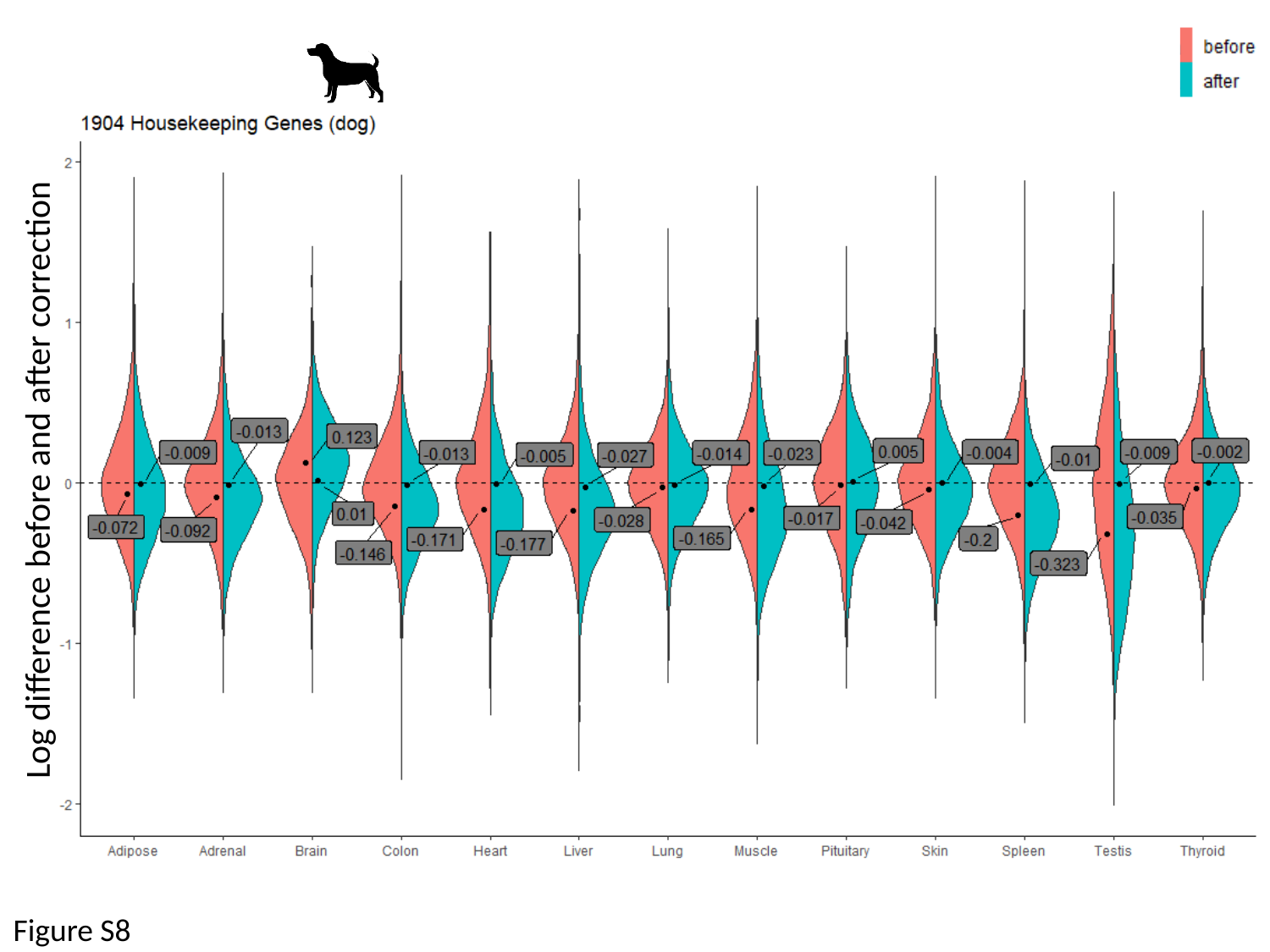

Log difference before and after correction
Figure S8

Supplement: Supplementary file 8 — Additional file 8: Figure S8 Distribution of 1,904 housekeeping and orthologous genes’ expression log difference before Trimmed Means of M values (TMM) correction and after TMM correction across 13 tissues in dog relative to human. The labeled values are the median of the distributions before and after TMM correction. The median of TMM corrected distribution is closer to 0, meaning that TMM correction makes the pre-clinical species distributions more comparable to human. [file 12859_2021_4414_MOESM8_ESM.pptx]

## Slide 1
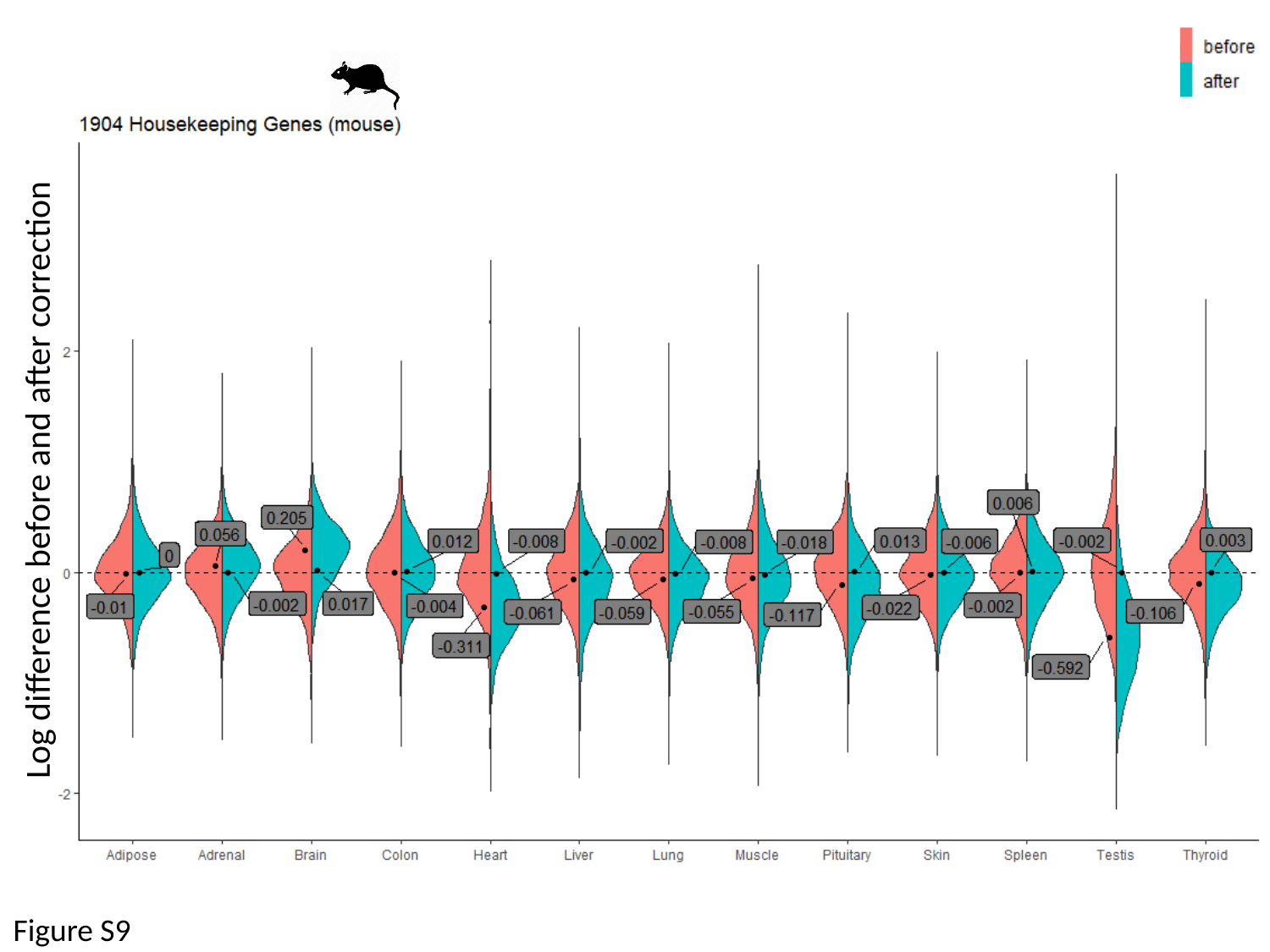

Log difference before and after correction
Figure S9

Supplement: Supplementary file 9 — Additional file 9: Figure S9 Distribution of 1,904 housekeeping and orthologous genes’ expression log difference before Trimmed Means of M values (TMM) correction and after TMM correction across 13 tissues in mouse relative to human. The labeled values are the median of the distributions before and after TMM correction. The median of TMM corrected distribution is closer to 0, meaning that TMM correction makes the pre-clinical species distributions more comparable to human. [file 12859_2021_4414_MOESM9_ESM.pptx]

## Slide 1
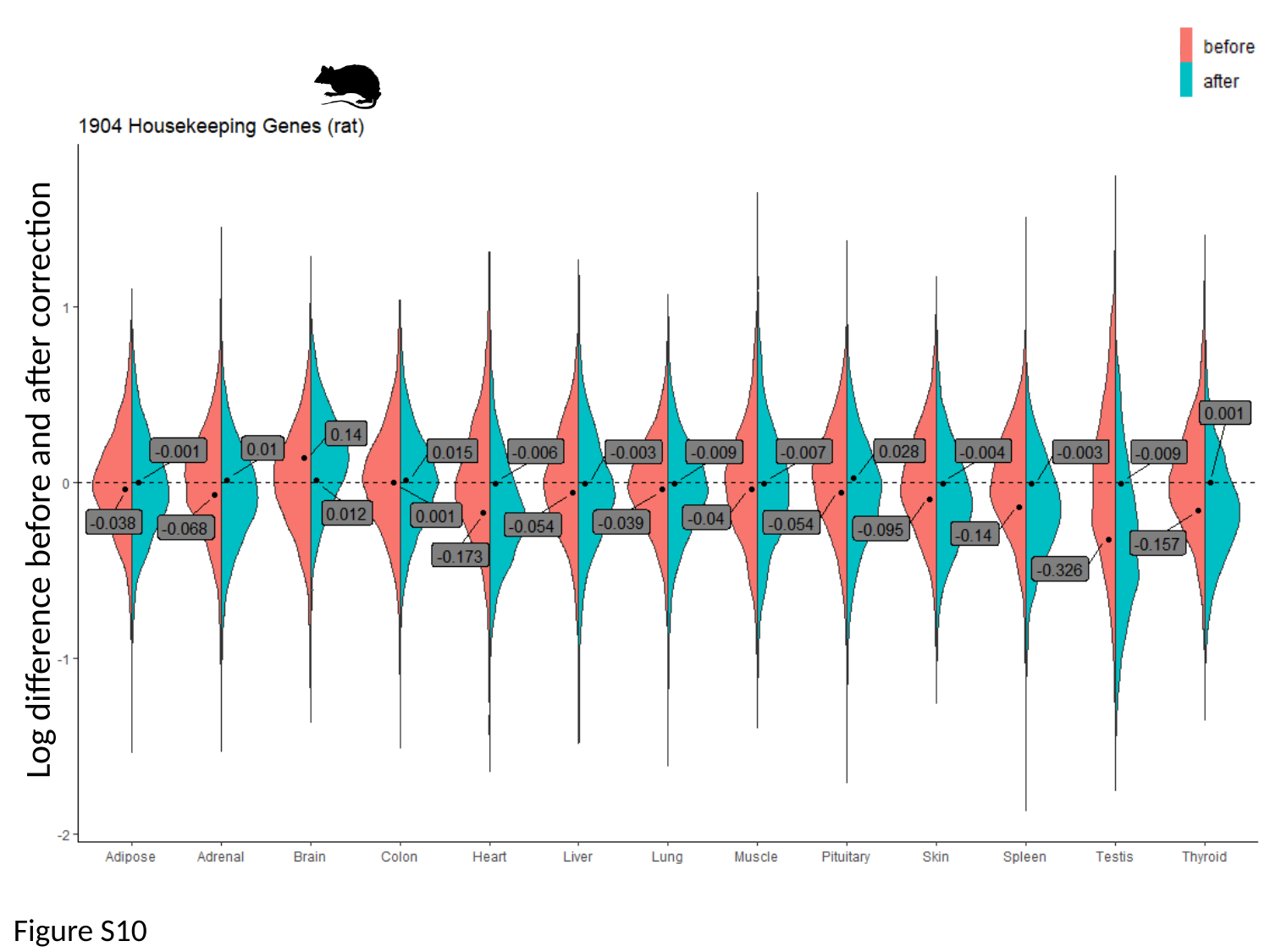

Log difference before and after correction
Figure S10

Supplement: Supplementary file 10 — Additional file 10: Figure S10 Distribution of 1,904 housekeeping and orthologous genes’ expression log difference before Trimmed Means of M values (TMM) correction and after TMM correction across 13 tissues in rat relative to human. The labeled values are the median of the distributions before and after TMM correction. The median of TMM corrected distribution is closer to 0, meaning that TMM correction makes the pre-clinical species distributions more comparable to human. [file 12859_2021_4414_MOESM10_ESM.pptx]

## Slide 1
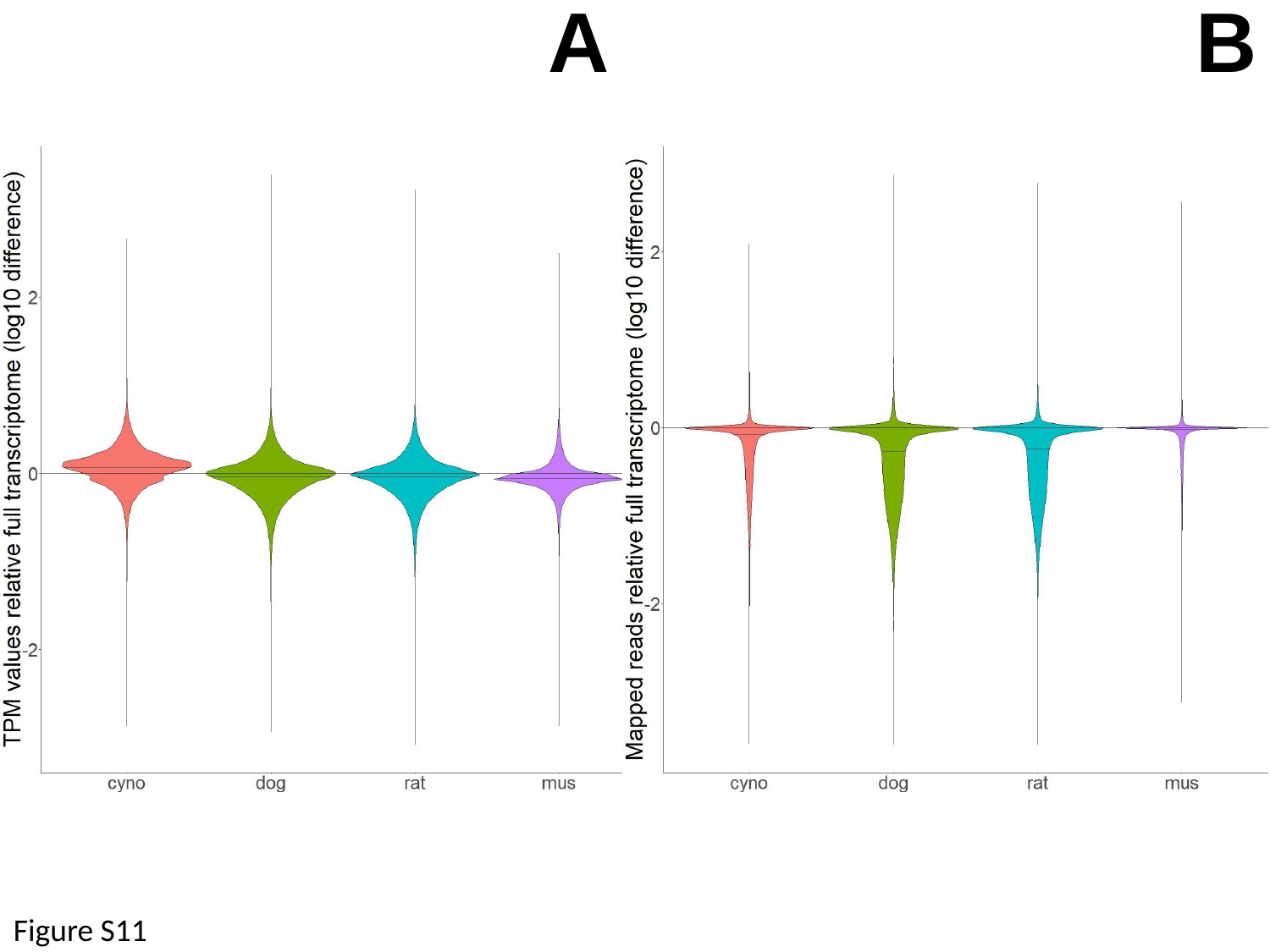

A
B
Figure S11

Supplement: Supplementary file 11 — Additional file 11: Figure S11 Both abundance and read mapping are reduced in pre-clinicalized human transcriptomes. A) tpm estimates are on average lower in pre-clinicalized transcriptomes for species, other than cyno. B) This is congruent with left skew in read counts compared to fully represented human transcriptome. [file 12859_2021_4414_MOESM11_ESM.pptx]

## Slide 1
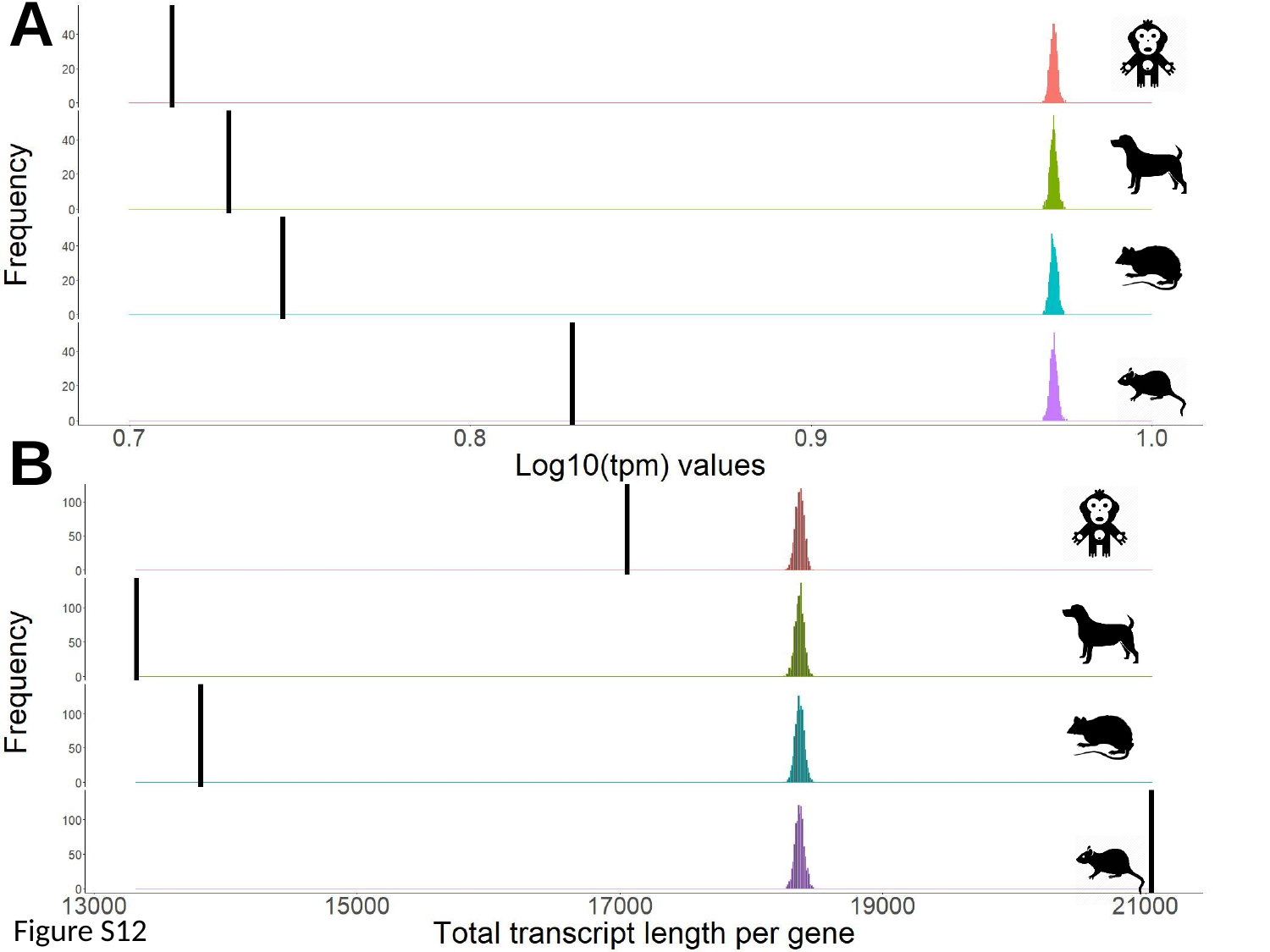

A
B
Figure S12

Supplement: Supplementary file 12 — Additional file 12: Figure S12 Inflation of tpm values in pre-clinicalized transcriptomes happens in genes with lower than average expression, while it is not related to gene length. A) Random subsampling of gene expression values (1000 permutations) of the same size as number of genes as ones found to have inflated tpm values in pre-clinicalized transcriptomes. The average expression in sampled genes is markedly higher for all species, compared to the average expression in the genes found to have inflated tpm in pre-clinicalized transcriptomes (black vertical bars). B) Same sampling did not yield a consistent difference between gene length of inflated tpm genes and the rest of the transcriptome. [file 12859_2021_4414_MOESM12_ESM.pptx]

## Slide 1
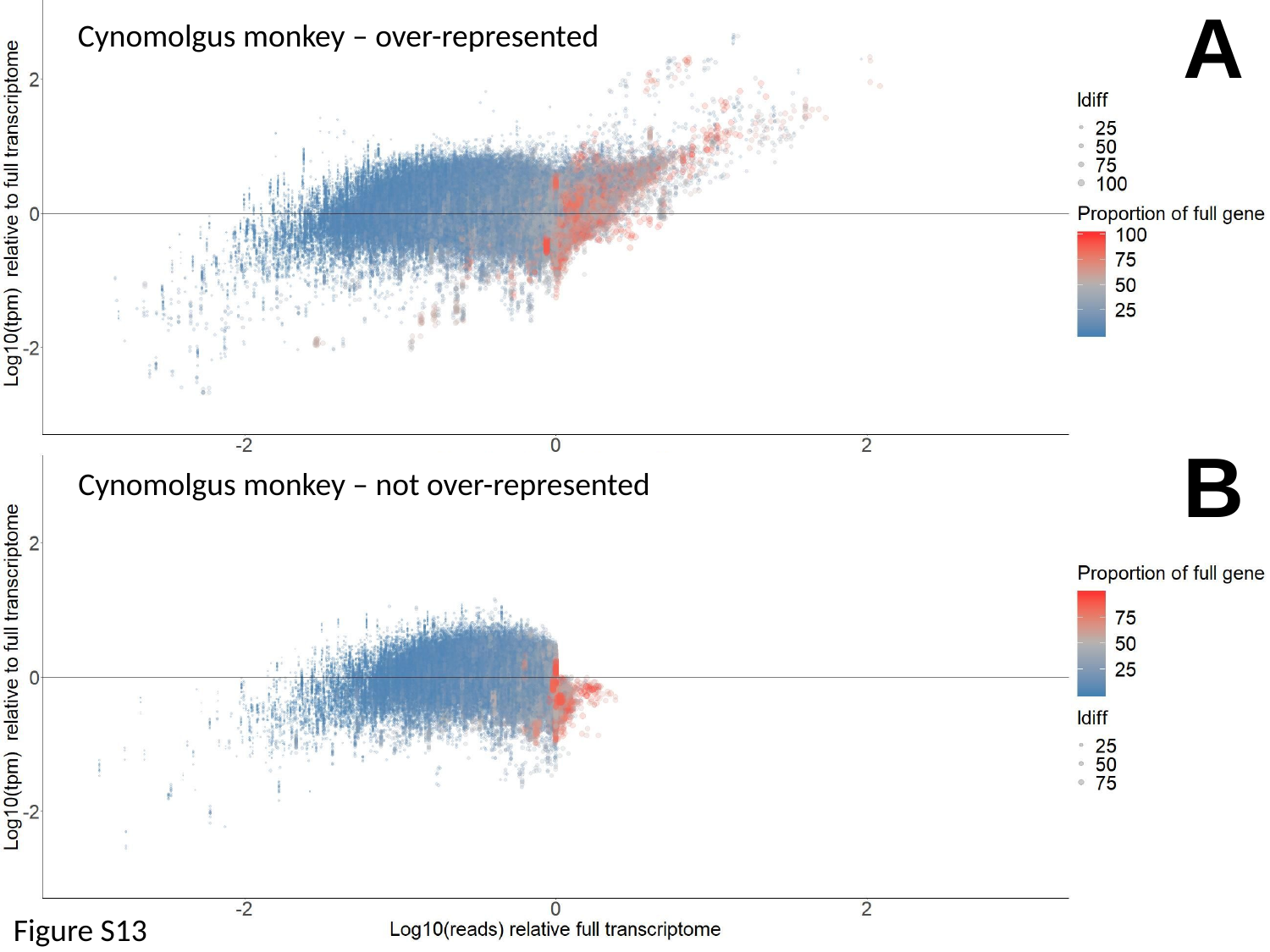

A
Cynomolgus monkey – over-represented
B
Cynomolgus monkey – not over-represented
Figure S13

Supplement: Supplementary file 13 — Additional file 13: Figure S13 Genes with inflated tpm values in pre-clinicalized transcriptome only experience such inflation, when large proportion of the total transcript length is present. A) Reveals genes that were shown to experience inflation in tpm when examined in a pre-clinicalized transcriptome. B) Same relationship for all other genes shows that inflation does not occur even when at high proportions of transcript length present. [file 12859_2021_4414_MOESM13_ESM.pptx]

## Slide 1
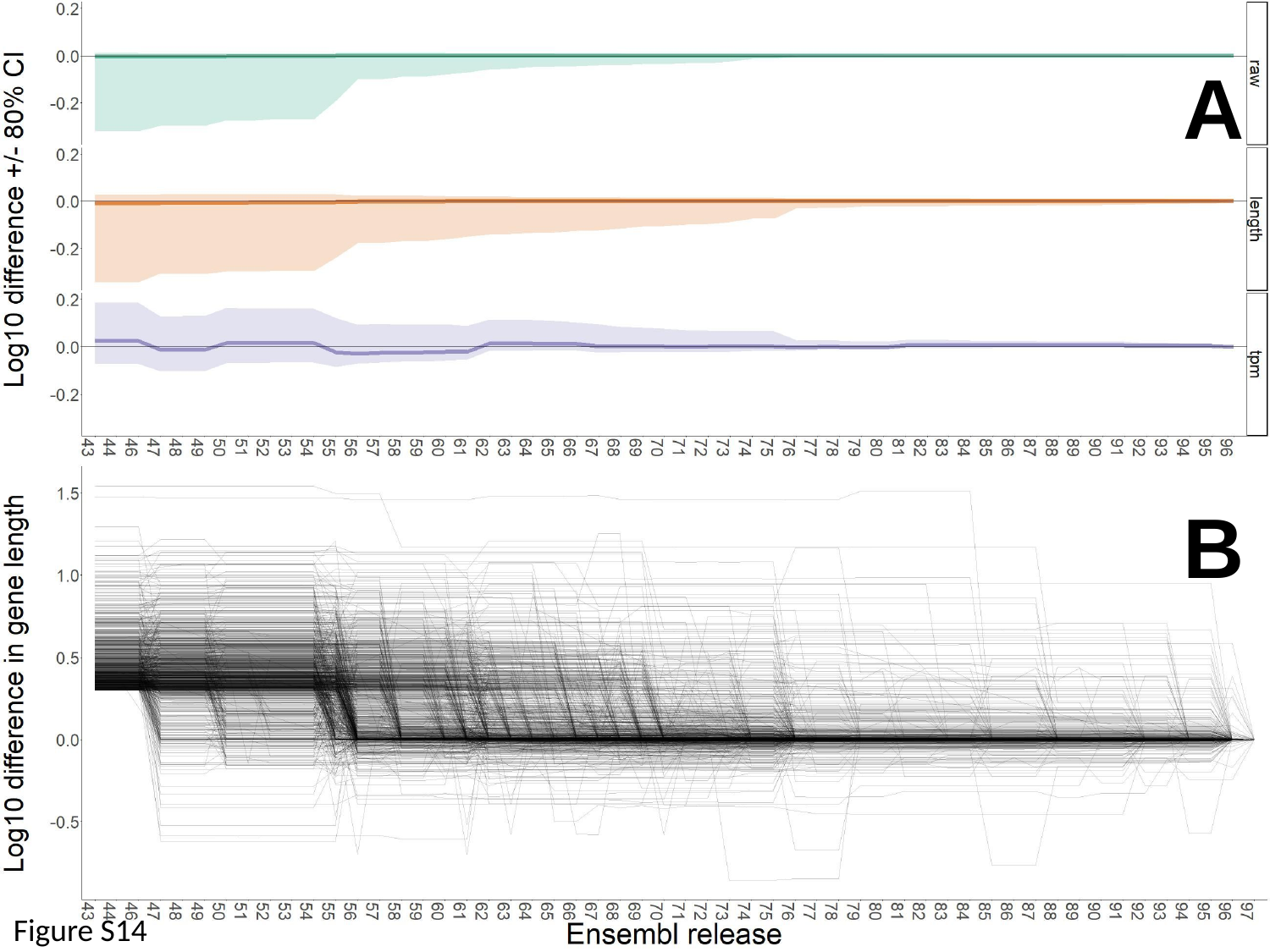

A
B
Figure S14

Supplement: Supplementary file 14 — Additional file 14: Figure S14 Historical variation in gene length plays a role in tpm estimates. A) In historical ensemble human transcriptome releases, genes that did not have inflated gene lengths have tpm estimates more consistent with ones from current day human transcriptome. B) Gene length variation in genes with historically inflated length estimates is highly variable even until very recent releases. [file 12859_2021_4414_MOESM14_ESM.pptx]
