# Supplementary material for: Annotation depth confounds direct comparison of gene expression across species
Source: BMC Bioinformatics. 2021 Oct 15;22:499. doi: 10.1186/s12859-021-04414-y (PMC8518172; doi:10.1186/s12859-021-04414-y)
Supplement: Supplementary file 2 — Additional file 2: Figure S2 Increases in relative normalized read counts per sample are consistent with reduction of tpm value across all examined orthologous genes. We examined all samples from Naqvi et al. [10], the GTEx and HPA datasets and focused on orthologous genes. We calculated the sum of gene length normalized raw reads per sample and related them to the most highly sequenced sample. We show a negative relationship (linear model) between log10(tpm) per gene values and total reads mapped across the entire orthologous transcriptome. [file 12859_2021_4414_MOESM2_ESM.pptx]

## Slide 1
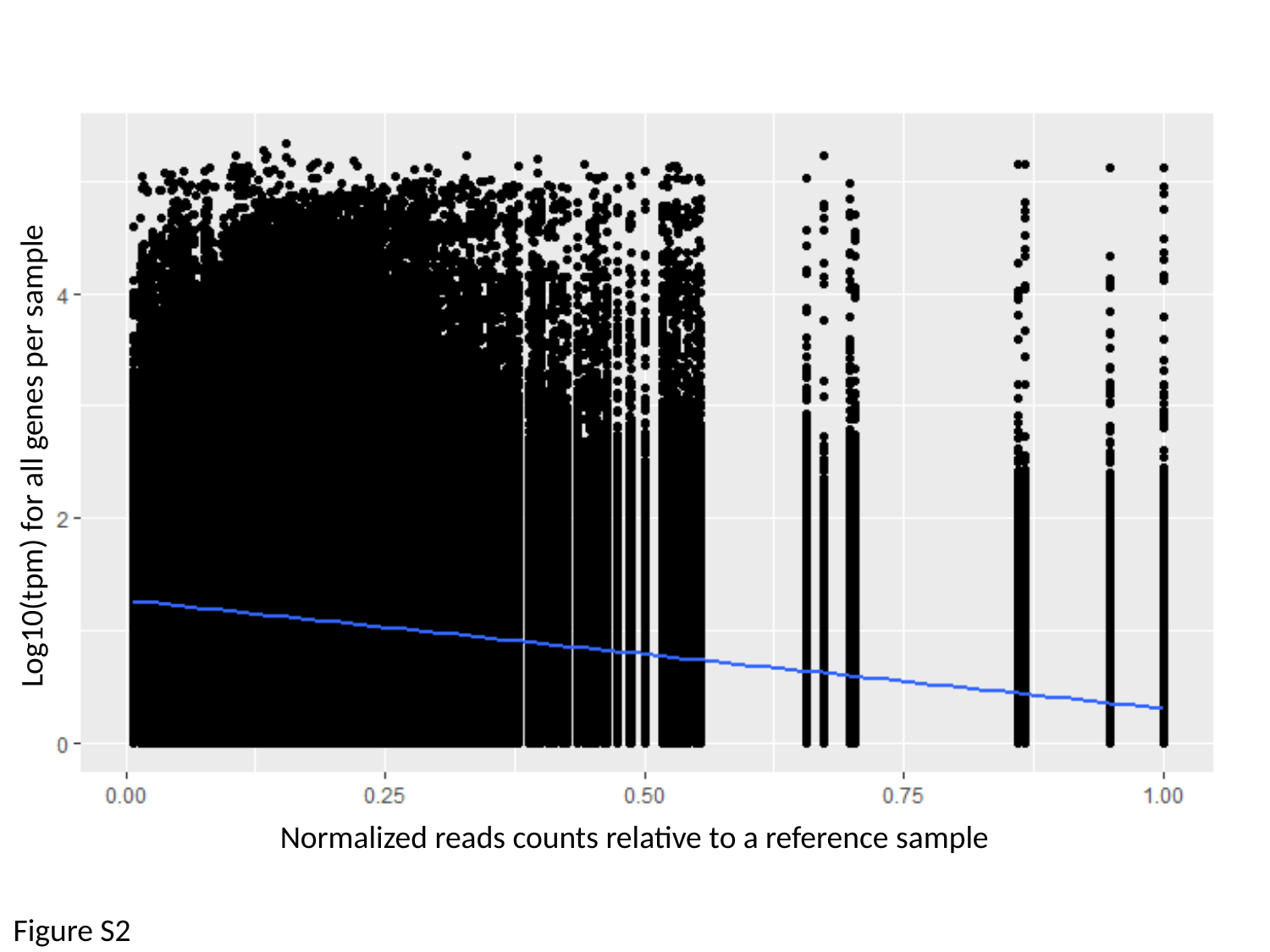

Log10(tpm) for all genes per sample
Normalized reads counts relative to a reference sample
Figure S2
